# Analysis of Copernicus’ ERA5 Climate Reanalysis Data as a Replacement for Weather Station Temperature Measurements in Machine Learning Models for Olive Phenology Phase Prediction †

**DOI:** 10.3390/s20216381

**Published:** 2020-11-09

**Authors:** Noelia Oses, Izar Azpiroz, Susanna Marchi, Diego Guidotti, Marco Quartulli, Igor G. Olaizola

**Affiliations:** 1 Vicomtech Foundation Basque Research and Technology Alliance (BRTA), 20000 Donostia, Spain; iazpiroz@vicomtech.org (I.A.); mquartulli@vicomtech.org (M.Q.); iolaizola@vicomtech.org (I.G.O.); 2 Agricolus s.r.l., 06100 Perugia, Italy; s.marchi@agricolus.com (S.M.); d.guidotti@agricolus.com (D.G.)

**Keywords:** phenophase, olive phenology modeling, BBCH scale, machine learning, base temperature

## Abstract

Knowledge of phenological events and their variability can help to determine final yield, plan management approach, tackle climate change, and model crop development. THe timing of phenological stages and phases is known to be highly correlated with temperature which is therefore an essential component for building phenological models. Satellite data and, particularly, Copernicus’ ERA5 climate reanalysis data are easily available. Weather stations, on the other hand, provide scattered temperature data, with fragmentary spatial coverage and accessibility, as such being scarcely efficacious as unique source of information for the implementation of predictive models. However, as ERA5 reanalysis data are not real temperature measurements but reanalysis products, it is necessary to verify whether these data can be used as a replacement for weather station temperature measurements. The aims of this study were: (i) to assess the validity of ERA5 data as a substitute for weather station temperature measurements, (ii) to test different machine learning models for the prediction of phenological phases while using different sets of features, and (iii) to optimize the base temperature of olive tree phenological model. The predictive capability of machine learning models and the performance of different feature subsets were assessed when comparing the recorded temperature data, ERA5 data, and a simple growing degree day phenological model as benchmark. Data on olive tree phenology observation, which were collected in Tuscany for three years, provided the phenological phases to be used as target variables. The results show that ERA5 climate reanalysis data can be used for modelling phenological phases and that these models provide better predictions in comparison with the models trained with weather station temperature measurements.

## 1. Introduction

In temperate ecosystems, temperature is a major driver of tree phenology [1], directly affecting crop productivity and fruit quality [2]. Therefore, gobal warming is expected to impact plant development and phenological shift, as well as agronomic practices. Because the fruit industry is labor intensive and the quality of production is time sensitive, appropriate planning of management interventions (e.g., spraying, harvesting) is key for generating climate-smart solutions for future agriculture. However, predicting phenological stages and interactions with fruit yield and quality is challenging, because inaccurate models are often used by farmers and technicians to address the impact of environmental conditions. Thus, if on one side the increased worldwide demand of quality products requires modernization of production systems, both at farm and industrial levels, on the other hand technological development calls for balanced cultivation technique and integrated production of fruit trees.

The olive tree (*Olea europaea* L.) and olive oil represent the most valuable economic, cultural and ecological elements in the Mediterranean region [3,4]. The prediction of olive flowering [5,6], yield prediction [3], as well as pests and diseases of this crop [7], can be modeled in order to determine the impacts of both environmental setting and agronomic management on olive tree productivity [8,9,10]. In olive tree, a relation between phenological phases and climatic variables was found [11,12], also associated with topographic features [13,14]. Modeling the occurrence of phenological stages is based on the accumulation of temperatures above a base temperature calculated on daily (growing degree day) or hourly (e.g., normal heat hours) time steps up to a fixed amount [15]. Growing degree day (GDD) is a measure of heat unit accumulation defined as temperature degrees above a base temperature, the base temperature being the temperature below where growing degree accumulation is irrelevant to phenological timing [16]. This information can be implemented in decision support frameworks for sustainable agronomic management of olive tree systems at various scales, as well as for promoting feasible adaptation strategies farming.

Models that are based on chilling accumulation or heat requirement are complex, and the effect of climatic factors, namely temperature, on reproductive phenology of olive trees has been poorly documented [17,18]. Indeed, tracking plant development based on the number of hours or days where the mean temperature is above a threshold value or within a given range of values is difficult in the highly fragmented cultivation area of olive trees throughout the Mediterranean environment [19]. Setting the onset of heat accumulation and the threshold for heat units varies with plant genotype and depends on weather data. Nevertheless, distance from weather stations (often in small number) to olive orchards (often of small size) may have a significant influence on microclimate, determining the spatial inconsistency of the meteorological datasets and the local inaccuracy of the model forecasts. Here, we explore the adequacy of substituting weather station temperature measurements with COPERNICUS ERA5 data by looking at the impact that this would have on the performance of the phenology phase prediction model. We compare a wide range of machine learning methods focused on the prediction of olive tree phenological development to select the best performer.

This study is part of the H2020 DEMETER project (http://h2020-demeter.eu), which focuses on the deployment of farmer-centric and interoperable smart farming-IoT (Internet of Things) based platforms. DEMETER’s pilots are used for evaluating the benefits from innovative interoperability mechanisms, monitor the evolution of stakeholder maturity, and support farmers in protecting the health and the quality of production. The pilot focusing on Decision Support System (DSS) to support modern olive tree growing aims to optimize irrigation and fertilisation in olive orchards, as well as integrated pest management, through an on-line platform (Agricolus OLIWES). The proposed platform integrates software, sensors and open data to provide farmers and technicians with complete and efficient assistance in the olive tree growing and olive oil production. The key factor for the effective development of this DSS is the use of machine learning to link local climate conditions with crop phenology predictions.

Marchi et al. [20] used a comprehensive regional phenology and agrometeorological database of the Tuscany Region (Italy), implementing a simple phenological model for the olive tree. Here, we linked the main phenological phases of olive trees in Tuscany with the phase-specific cumulative GDD. We applied a new methodology in order to optimise the base temperature used in the calculation of GDD in order to improve predictions of olive tree phenology. Based on the machine learning approach, we tested whether replacing weather station temperature measurements with Copernicus ERA5 temperature data was suitable in terms of performance of the phenology phase prediction models. To this aim, accurate phenology phase prediction models were developed and the base temperature used in the calculation of GDD optimised in order to make better predictions of olive tree development.

## 2. Materials and Methods

### 2.1. Phenology Prediction Model

#### 2.1.1. Model Inputs

GDD and DOY were used as the model inputs. DOY is the number of days since the 1st of January of each year. GDD is the accumulation of temperature degrees above a base temperature, the base temperature being the temperature below which vegetation ceases to be biologically active. A default base temperature of 10 °C was used for GDD calculation in all cases, except in the base temperature optimisation section (Section 3.4). For olive tree phenology, base temperature 10 °C is almost a standard (e.g., [7,20]), even if it is possible to find studies in which lower base temperatures are used (e.g., 5 °C [21]). The first day for starting heat accumulation is considered the 1st of January.

There are various ways in which GDD can be calculated [22]. The simplest calculation deduces the base temperature from the average of the daily maximum and minimum temperatures. This has been denoted as *GDD Tavg* in this study. This calculation disregards the fact that the plant could have been exposed to temperatures that are above the base temperature during the day, even if the average between the maximum and minimum daily temperature is not above the base temperature.

Following the Allen method [23], GDD are calculated using the single sine method to approximate hourly temperatures, given the daily minimum and maximum temperatures, a base temperature and no upper cutoff. The hourly accumulation of temperature degrees above the base temperature provides the degree-hours and the daily average of degree-hours is the GDD. This method assumes that the temperature curve is symmetrical around the maximum temperature. GDD calculated following this method has been denoted as *GDD Allen*.

#### 2.1.2. Target Variable

The target variable was the phenological phase, as described in the BBCH-scale (Biologische Bundesanstalt, Bundessortenamt und Chemische Industrie) for olive trees. The BBCH-scale uses numbers to label the different phases but it is not a numerical variable and the distances between the numeric labels bear no relevance. Thus, a ranking of the BBCH-scale for olive trees has been generated and a new variable that gives the rank of the development label for each data point is created. The latter rank variable is used as the target variable in the prediction models. When regression models are used, the output is discretised (e.g. with floor, round, ceiling functions) in order to obtain an integer value.

### 2.2. Baseline Model

A baseline model [20] was used to benchmark the performance of the machine learning models tested. This baseline model is very simple: for each phenology phase, a specific GDD accummulation is considered. The baseline model works as follows. First, the threshold for each BBCH phase is set to the average GDD for all the data points in the training set with this BBCH phase. Subsequently, given a GDD value, take as the prediction the BBCH phase (its rank, really) for which the given GDD is greater than or equal to its threshold, but lower than the threshold for the next phase.

### 2.3. Data Used

This section presents the different sources and types of data that were used for this research.

#### 2.3.1. Phenology and Weather Station Data

The data that were used in this research came from phenology monitoring activity in olive orchards in Tuscany (North-Central Italy). Tuscany is characterized by relatively marked climatic variability due to the presence of the Apennine chain (North-Eastern border), inner hills, and river valleys with East-West orientation. Whereas the typically mild Mediterranean climate characterises coastal areas, conditions resembling those of continental areas may occur inland. The annual mean temperature is about 14 °C, while the total annual precipitation varies from a minimum of 600 mm in the central and southern coastal areas to 2000–3000 mm in the Apuan Alps [24].

Monitoring activity was carried out by trained technicians in selected olive orchards in eight provinces, across the regional territory. Tuscany is one of the most important regions in Italy for the production of high quality olive oil [25]. The monitored olive orchards were located in the most suitable areas for olive oil production, representing both coastal and inland conditions, and they were traditionally managed.

Phenological observations were done in 2008–2010 (three years). For each olive orchard, five olive trees in good health conditions and with homogenous productivity were selected, in the core area of the orchard, excluding those at the edge. The monitored olive trees were all belonging to the Frantoio cultivar. The olive orchards were selected for their closeness to one of the agrometeorological stations of the regional network. A simplified BBCH scale was used in order to assess the phenological stage for each observation. The monitoring protocol consisted in visiting the orchards at different time span during the year, according to the developmental rate of olive trees in the different seasons. Weather data were obtained from 19 different weather stations of the Tuscany Regional agrometeorological network. One agrometeorological station of the regional monitoring network was close to each of the olive orchards. The distance from the agrometeorological station and the monitored olive orchard varied from 0 (in-farm station for the majority of olive orchards) to 16 km.

**Figure 1:**
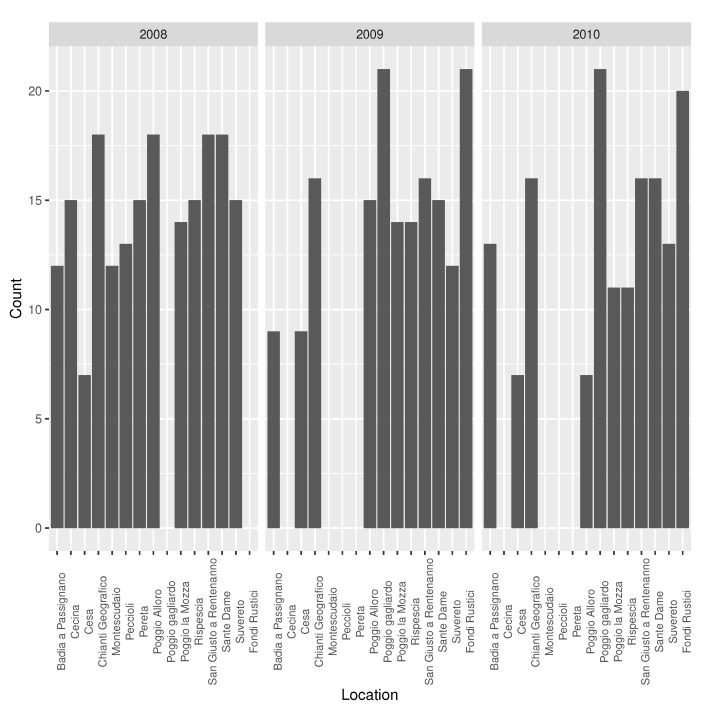
Dataset size by location and year.

There are 822 data points in the dataset that belong to 22 different locations with an average of 34 data points per location and an average of 274 data points per year (Figure 1).

#### 2.3.2. Copernicus’ Era5 Climate Reanalysis Data

This research uses COPERNICUS’ ERA5 data [26] thta were obtained from Google Earth Engine [27]. The ERA5 dataset is the fifth generation ECMWF atmospheric reanalysis of the global climate [28]. Reanalysis combines model data with observations across the world into a globally complete and consistent dataset. ERA5 replaces its predecessor, the ERA-Interim reanalysis. ERA5 DAILY provides aggregated values for each day for seven ERA5 climate reanalysis parameters, among which is the 2 m air temperature. Additionally, the daily minimum and maximum air temperature at 2 m has been calculated based on the hourly 2 m air temperature data. ERA5 data are available from 1979 to three months from real-time. More information and more ERA5 atmospheric parameters can be found at the Copernicus Climate Data Store. This research uses the daily minimum and maximum air temperature at 2 m from the ERA5 data.

The ERA5 data cover the Earth on a 30 km grid and resolve the atmosphere while using 137 levels from the surface up to a height of 80 km, whereas the agrometeorological stations provide scattered weather data, with incomplete spatial coverage, being scarcely useful as a source of information for the implementation of predictive models.

### 2.4. Ml Model Evaluation and Selection

The models that have been tested are linear regression, classification and regression trees, random forests, artificial neural networks, stochastic gradient boosting, and extreme gradient boosting (linear and tree). Linear regression [29] is a linear approach to modeling the relationship between a dependent variable (the phenological phase in this case) and one or more explanatory variables (i.e., DOY and GDD). Classification and regression trees (CART) use decision tree learning [30], a predictive modelling approach that is used in machine learning. A decision tree, as a predictive model, goes from observations about an item (represented in the branches) to conclusions about the item’s target value (represented in the leaves). Decision trees are popular, because they are simple and intelligible. A random forest (RF) [31] is an ensemble method that operate by training many decision trees and outputting the class that is the mode of the classes (classification) or mean prediction (regression) of the individual trees. An artificial neural network (ANN) is a machine learning method vaguely inspired by the biological neural networks that constitute animal brains. A collection of connected nodes form the ANN. When a node receives a signal it processes it and transmits the signal to other neurons. The output of each neuron is computed by some non-linear function of the sum of its inputs. Stochastic gradient boosting (GBM) [32] constructs additive regression models by sequentially fitting a simple base learner using a subsample of the training data drawn at random (without replacement) from the full training dataset. It generalizes the ensemble model by optimization of an arbitrary differentiable loss function. Extreme gradient boosting (XGBoost) [33] is an implementation of gradient boosting designed for speed and performance. In this paper, XGBoost for linear models and decision trees was used. XGBoost gained popularity due to its good performance with structured or tabular data.

Each model was used twice, one time with linear features and another time with polynomial features up to the fourth degree. In the different sections (feature selection, model selection, and base temperature optimisation), the dataset is repeatedly split (100 times in each section) into training (70%) and test (30%) disjoint sets with the particularity that the data have been chosen using stratified sampling for the different years and locations. The training set is used for model tuning and training of the final model. R’s Caret [34] package’s *train* method with three repeats of 10-fold cross-validation was used for parameter tuning and model training. It might be possible to achieve better performance by customising the hyperparameter optimisation and fine-tuning of the models. The different models were evaluated on the test set according to accuracy and root-mean-square error (RMSE) metrics. Even though, strictly speaking, this is a classification problem, we have applied both regression and classification algorithms and, in addition to accuracy, we also used the RMSE metric of regression, because the classes are strictly ordered and RMSE correctly penalises higher deviances of the predicted value from the observed value more than lower deviances. The objective was two-fold: to correctly classify as many cases as possible and, for those that are not correctly classified, for the prediction to be as close to the ground-truth as possible. We propose the following combination metric to unite this two-fold objective: the product of the RMSE and the complement of the accuracy (or error rate) (see Equation (1)). The objective was to minimize the combined metric.

(1) Combinedmetric=(1−Accuracy)×RMSE

### 2.5. Base Temperature Optimisation

Following the lessons that were learnt in [35], to optimise the value of the base temperature to maximise the accuracy and to minimize the RMSE, only test values that were less than or equal to 10 °C for base temperature have been used. Specifically, the temperature values tested are those from 0 °C to 10 °C with a step of 0.5 °C. In order to estimate the mean value for a metric (accuracy, RMSE, or combined metric) of a model, given a base temperature, a sample from the population was selected and the confidence interval for the population mean was calculated. In other words, for a given model and base temperature, a sample containing 100 observations of accuracy and RMSE was created. Each observation was randomly generated independently from the values of the other observations by randomly partitioning the dataset into training (70%) and test (30%) sets, training the model on the training set, and calculating the metrics on the test set.

## 3. Results

### 3.1. Weather Station and Era5 Data Comparison

#### 3.1.1. Gdd Calculation Comparison

GDD was calculated with the daily maximum and minimum temperature coming from both weather station measurements, denoted as *GDD Tavg* and *GDD Allen*, and ERA5 data, denoted as *ERA5 GDD Tavg* and *ERA5 GDD Allen*.

##### GDD Tavg

**Figure 2:**
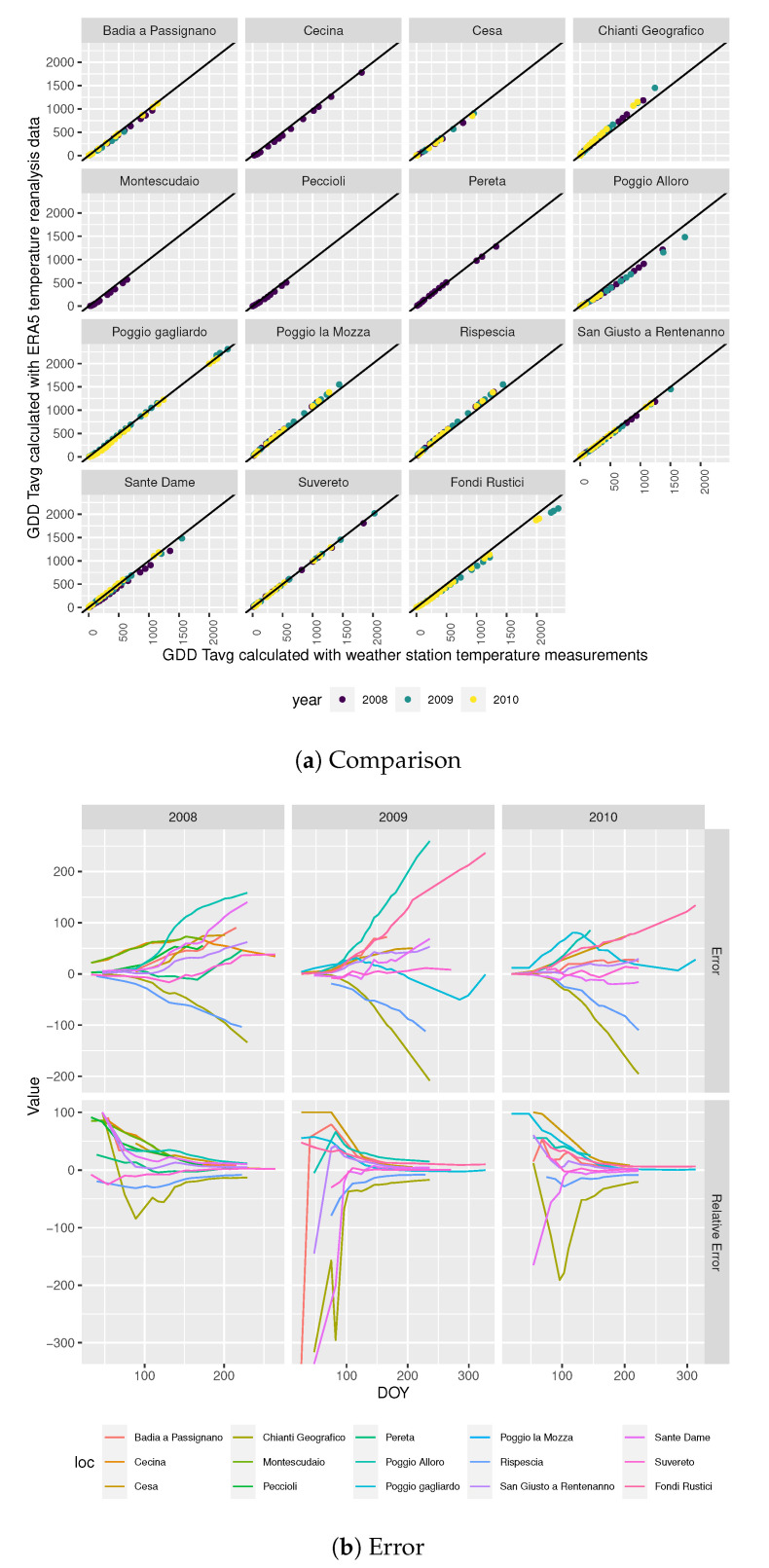
*GDD Tavg calculation*.

Figure 2a shows the relationship between *ERA5 GDD Tavg* and *GDD Tavg*, with the solid line marking the identity. The figure shows that the two values are closer for some locations than for others. For example, for Suveretto the points fall on the identity line, but for other locations, such as Poggio Alloro, the points are off the identity line.

Figure 2b shows the error in the *ERA5 GDD Tavg* calculation taking, as reference, the *GDD Tavg* value by DOY. The figure shows that the error does not cancel out as the DOY increases. Indeed, Figure 2a shows that for a given location the *ERA5 GDD Tavg* tends to be always above or below the reference value. However, looking at the relative error with respect to the reference value, it can be seen that the relative error converges to a fixed percentage that changes by location.

##### GDD Allen

**Figure 3:**
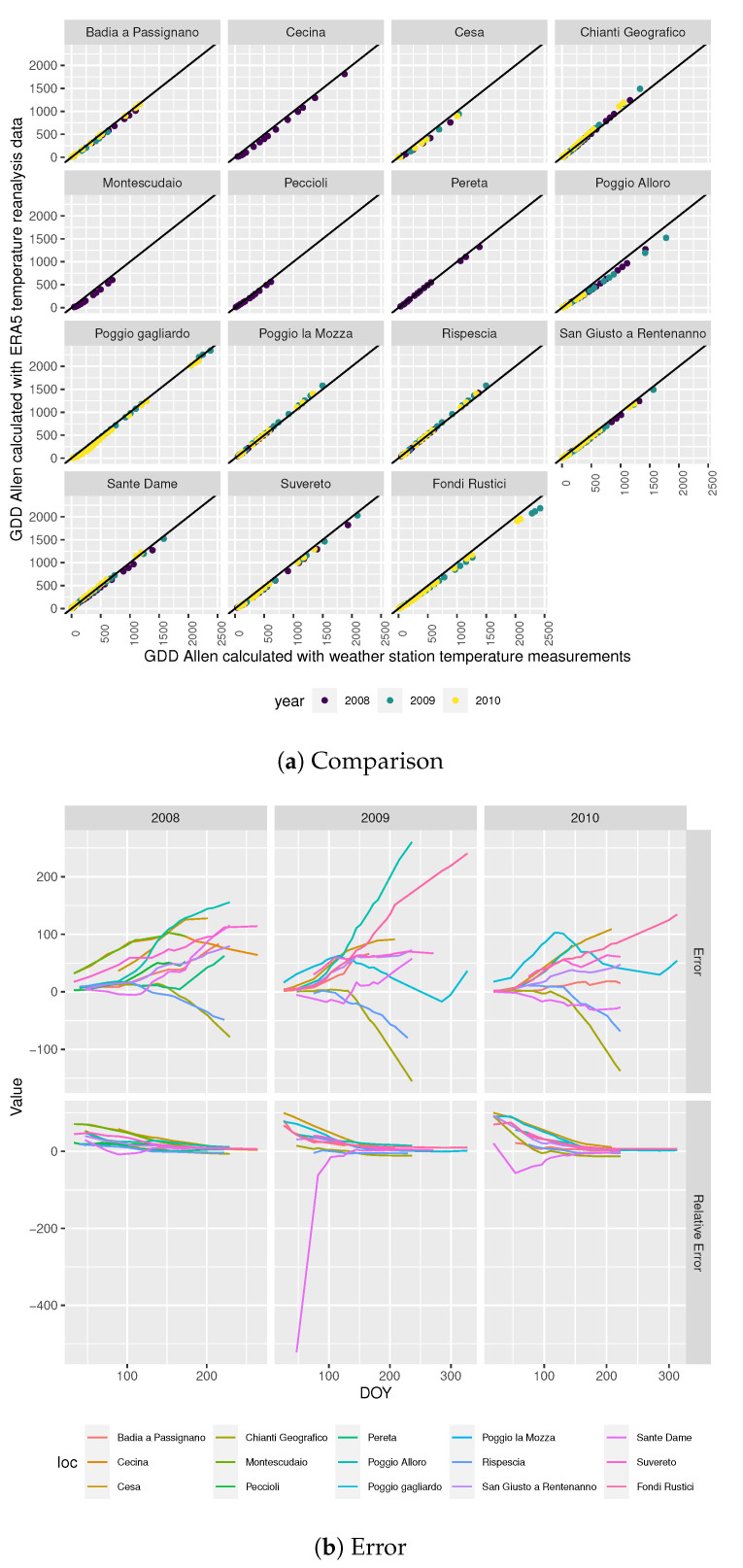
*GDD Allen calculation*.

Similarly to what happens with *GDD Tavg*, Figure 3a shows that *ERA5 GDD Allen* is closer to *GDD Allen* for some locations than for others. However, in this case, Figure 3b shows that the relative error in the *ERA5 GDD Allen* calculation with respect to the *GDD Allen* value is smaller than the relative error that is presented in Figure 2b and it also converges to a fixed percentage that changes by location.

#### 3.1.2. Predictor Performance Comparison

In order to compare the performance of different feature subsets as predictors of phenology phase in prediction models, a random forest (RF) model was used because it has good performance in genera [35]. *GDD Tavg* and *GDD Allen*, calculated both with the weather station temperature measurements and with the ERA5 temperature reanalysis data, and DOY were the possible model inputs.

**Figure 4:**
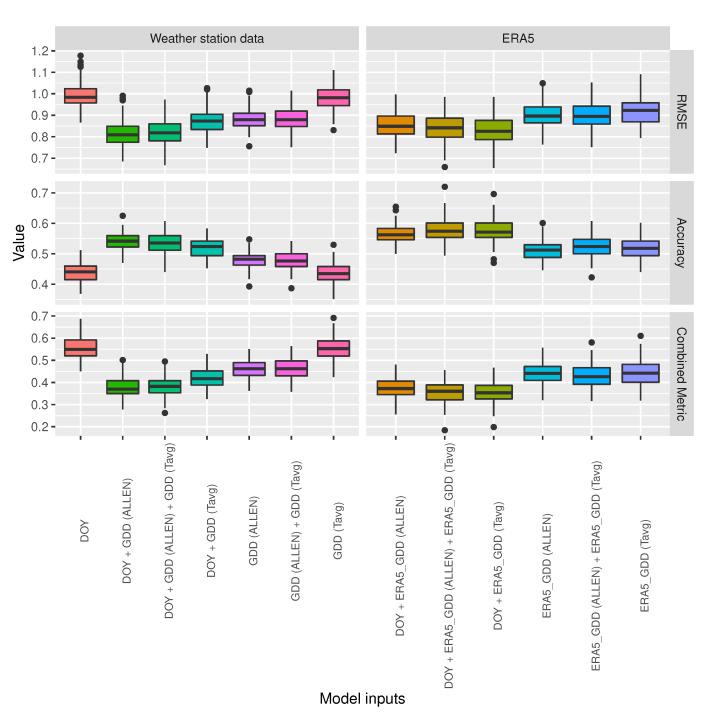
Random forest model performance comparison using different predictors.

Figure 4 shows the performance of random forest models while using different predictors for 100 different training-test set splits of the data. The predictors have been classified into two groups: ‘Weather station data’ are the feature sets that contain only features that were obtained from weather stations temperature measurements and ‘ERA5’ is the group of feature sets that contain only features calculated from the ERA5 dataset. The first observation drawn from Figure 4 is that DOY on its own is not a good predictor but, within the same group, those feature sets that contain DOY perform better than the feature sets without it, they have higher accuracy, lower RMSE, and lower combined metric. Accordingly, it is important to have DOY as one of the predictors. In the ’Weather station data’ group, the feature set consisting of (DOY, *GDD Allen*) is clearly the best performer with higher accuracy, lower RMSE, and lower combined metric than the other feature sets in the same group. In the ’ERA5’ group, the feature set consisting of (DOY, *ERA5 GDD Tavg*) is the best performer with lower RMSE and lower combined metric and similar accuracy to the feature set that includes the previous two features and also *ERA5 GDD Allen*. Remarkably, ERA5’s (DOY, *ERA5 GDD Tavg*) feature set outperforms weather station data’s (DOY, *GDD Allen*) feature set in terms of combined metric, having clearly higher accuracy and only slightly higher RMSE.

**Figure 5:**
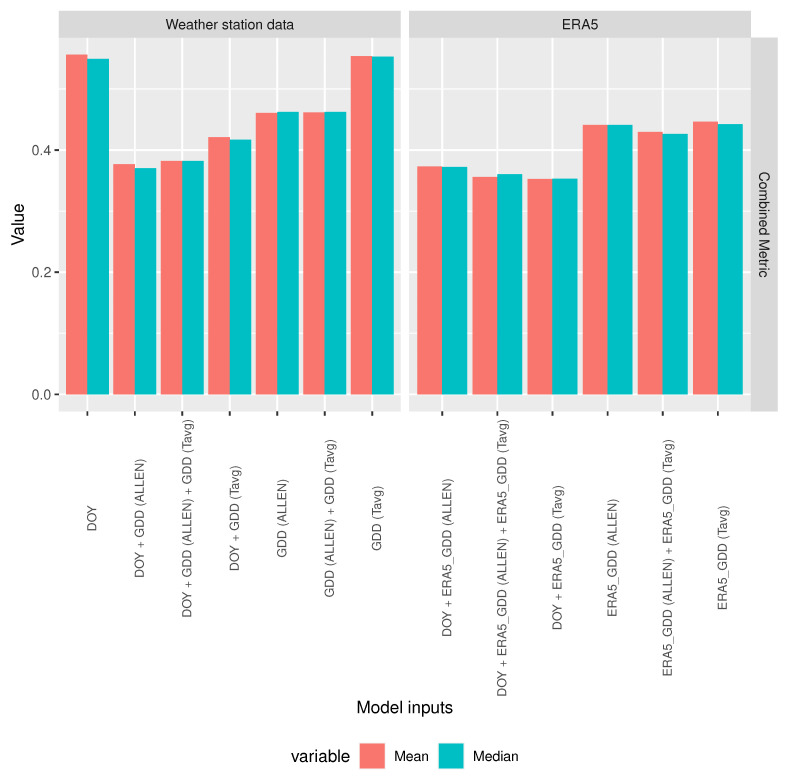
Combined metric mean and median values for random forest model performance comparison using different predictors.

Figure 5 shows the mean and the median of the combined metric for the different feature sets. ERA5’s (DOY, *ERA5 GDD Tavg*) is the overall best performer of the two groups, in both mean and median.

### 3.2. ML Model Selection

Table 1 describes the two different scenarios for which the ML model selection process was carried out. Each scenario corresponds to one of the predictor groups defined in Section 3.1.2 and, in each predictor group, only the best performing predictor combination was taken into account.

**Figure 6:**
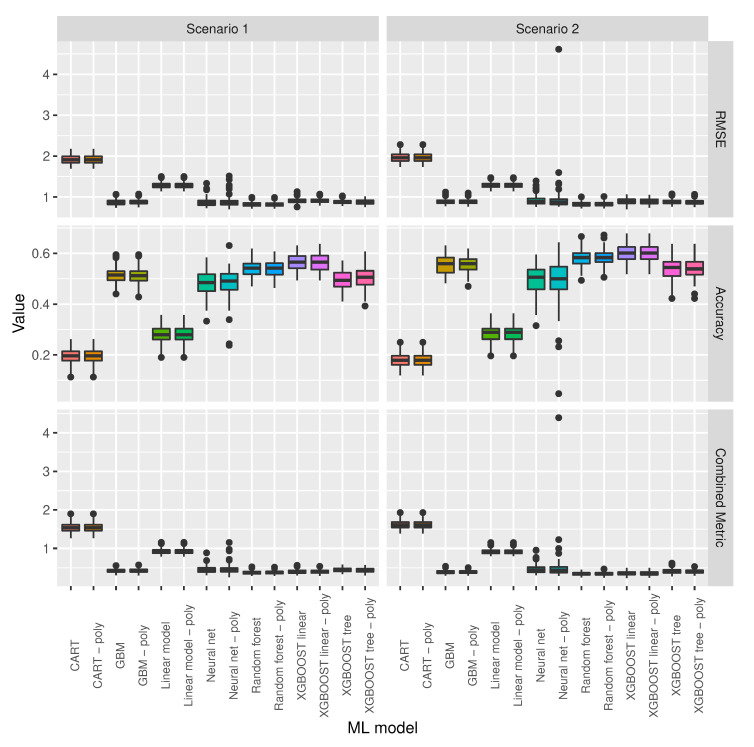
Performance metrics for different ML models trained and tested under the scenarios specified in Table 1.

Figure 6 shows the performance metrics for different ML models trained and tested under the scenarios specified in Table 1. This figure shows that three models can be rejected without further consideration for this dataset. These models are CART, linear regression, and neural nets.

**Figure 7:**
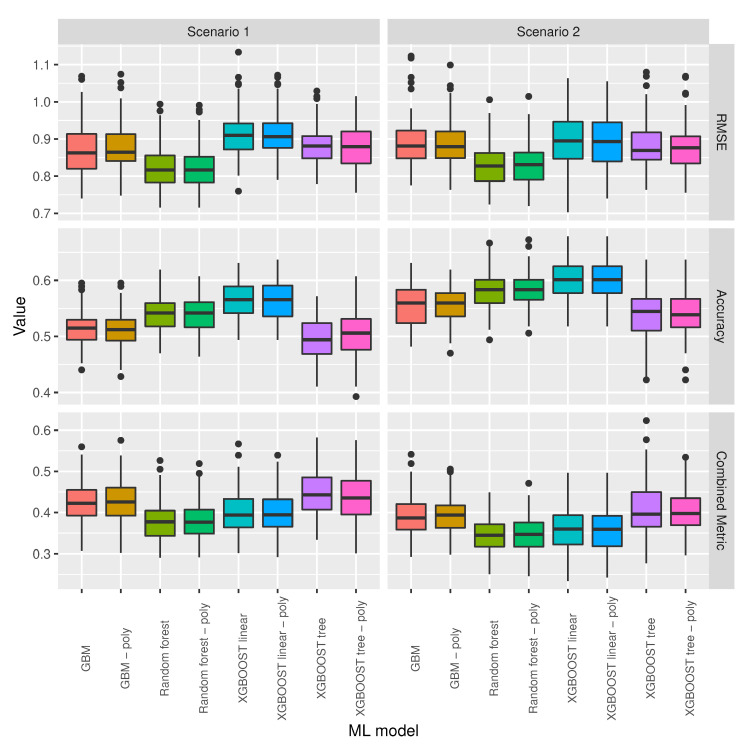
Model selection for the scenarios specified in Table 1.

Figure 7 shows the same information as Figure 6, but with the three worst performing models removed in order to allow for a better comparison of the remaining models. The figure shows that XGBoost linear and XGBoost linear with polynomial features are the two models with highest accuracy and that random forest and random forest with polynomial features are the two models with lower RMSE and lower combined metric in the two different scenarios.

**Figure 8:**
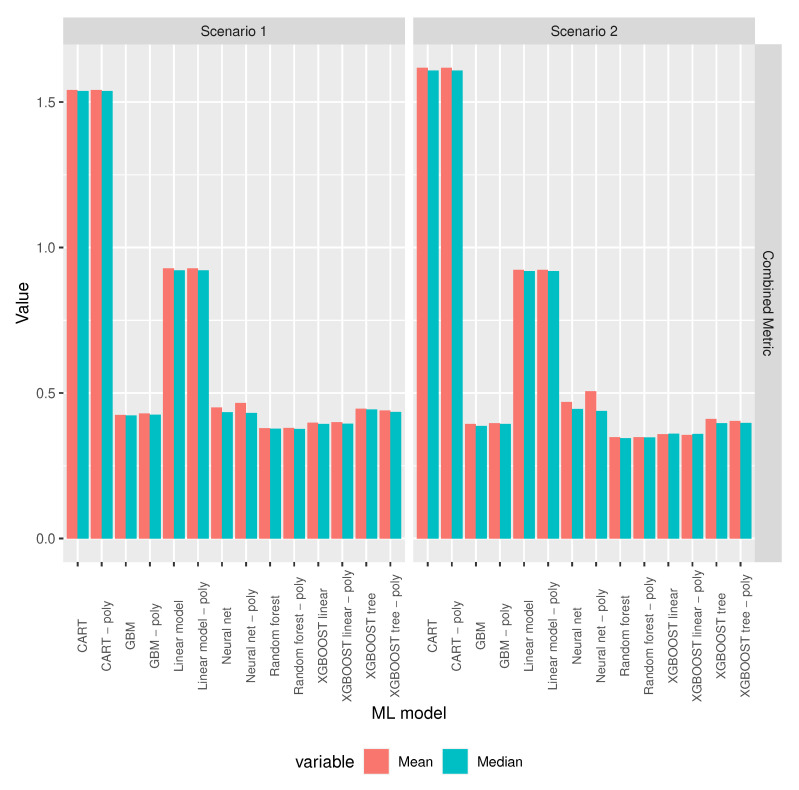
Combined metric mean and median values for the scenarios specified in Table 1.

When taking into account that there were models that had outlier values for accuracy and RMSE, we used the mean of the combined metric as the model selection criterion: for each selected feature combination, the model with the minimum mean combined metric was the selected model for the corresponding feature set. Figure 8 shows that random forest and random forest with polynomial features have very similar means, followed by XGBoost linear and XGBoost linear with polynomial features, with the means of these four models well below the means of the remaining models.

Table 2 shows the best performing models for each scenario in Table 1 with the value for the mean combined metric shown numerically.

### 3.3. Baseline and Selected ML Models’ Comparison

This section presents a comparison of the selected ML models against the baseline model described in Section 2.2. Table 3 summarises the three different scenarios in this comparison. The first two are the scenarios that are defined in Table 2 and the third scenario is the baseline model.

**Figure 9:**
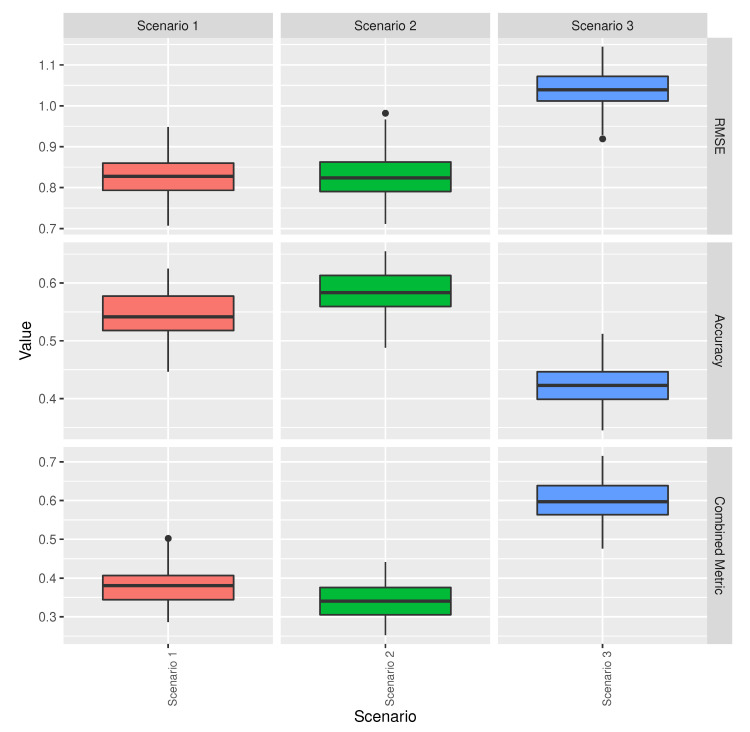
Metrics’ comparison for the models in the scenarios described in Table 3.

Figure 9 compares the metrics for the different models in the scenarios described in Table 3. It shows that the selected ML models have higher accuracy and lower RMSE than the baseline model and that scenario 1 has lower accuracy and similar RMSE to scenario 2. Therefore, it can be concluded that scenario 1 performs worse than scenario 2. In other words, as far as accuracy and error are concerned, it is advisable to use a well-tuned ML model with ERA5 temperature reanalysis data, instead of using the baseline model or a ML model with weather station temperature measurements.

**Figure 10:**
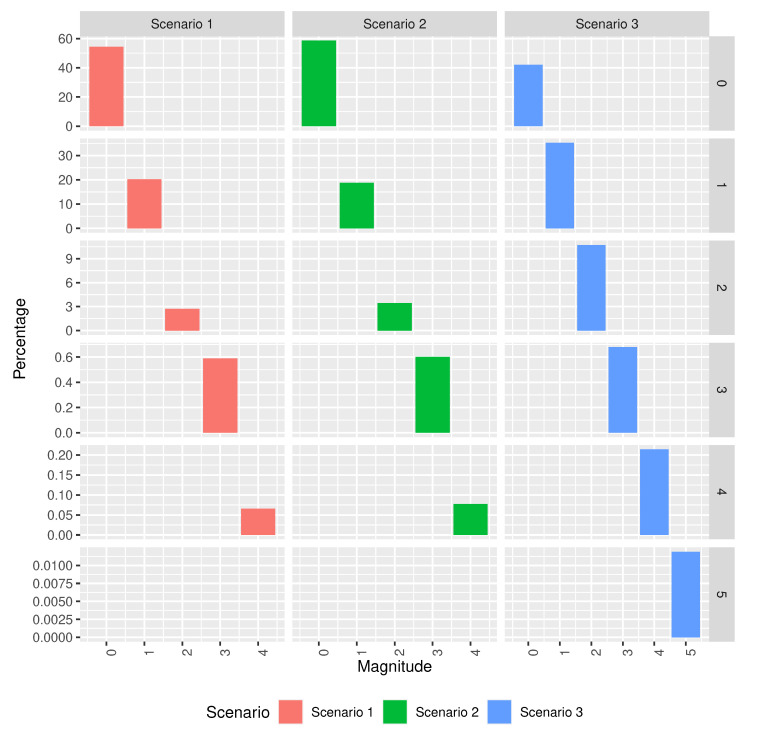
Residual histogram for the models in the scenarios described in Table 3.

Figure 10 compares the histograms of the magnitudes of the residuals (i.e., absolute value of the residuals) for the different models in the scenarios described in Table 3. It shows that scenarios 1 and 2 (i.e., the ML models selected) have better accuracy than scenario 3 (i.e., the baseline model), which is, the percentage of residuals of magnitude 0 is greater for the ML models than for the baseline model. Scenario 3 is the only scenario to have residuals of magnitude 5 and it is also the one with a significantly larger number of residuals of magnitude 4. All in all, scenario 3 is the worst performing scenario with more and larger errors. Scenario 1, on the other hand, has lower accuracy than scenario 2 but most of its residuals are of magnitude 1 and has a lower number of residuals of magnitude greater than 1 than scenario 2.

**Figure 11:**
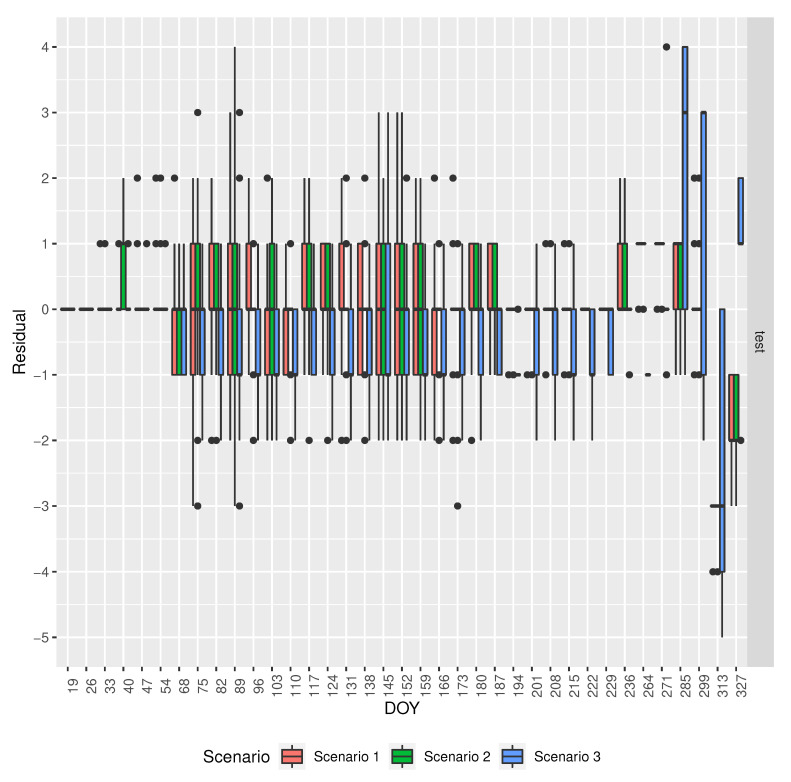
Comparison of the residuals by DOY for the different models’ in the scenarios described in Table 3.

Figure 11 shows the residuals by DOY for the different models in the scenarios that are described in Table 3. It shows that scenario 3, i.e., the baseline model, has the largest magnitude errors for large DOYs, whereas, for smaller DOYs, it tends to underestimate the phenology phase. In the light of this information, it might be interesting to create an ensemble model combining the baseline model with each of the models in the other scenarios and look at the performance of these ensembles.

**Figure 12:**
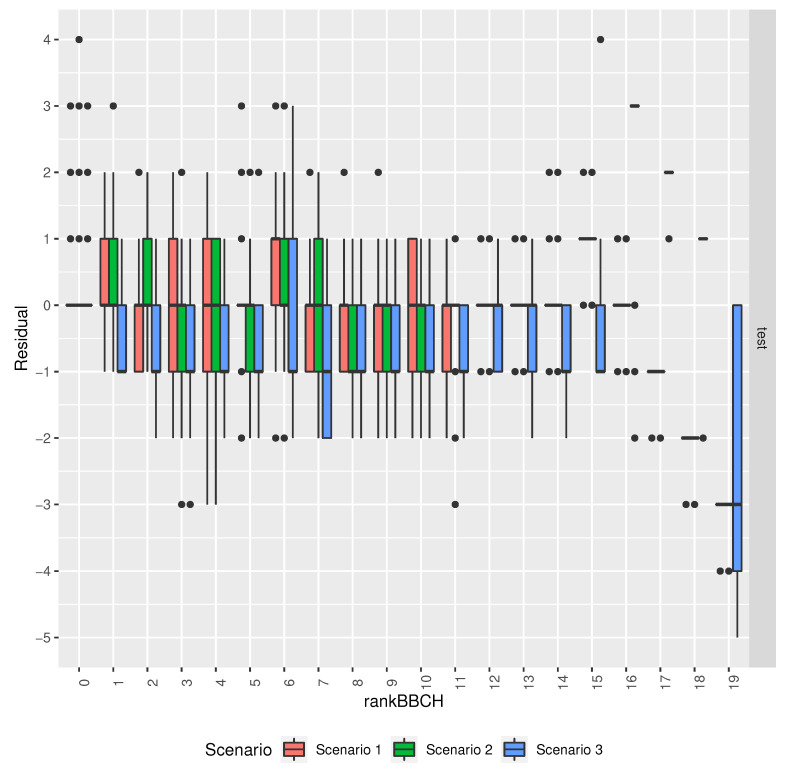
Comparison of the residuals by target output for the different models’ in the scenarios described in Table 3.

Figure 12 shows the residuals by target output for the different models in the scenarios that are described in Table 3. It shows that, in the first 16 phenology phases, the baseline model tends to underestimate the phase, while the models in the other scenarios have better accuracy for these phases. It also shows that the baseline model makes errors of large magnitude for the last four phenology phases, often overstimating the phase, and that the models in scenarios 1 and 2 understimate the phase in those same phases.

**Figure 13:**
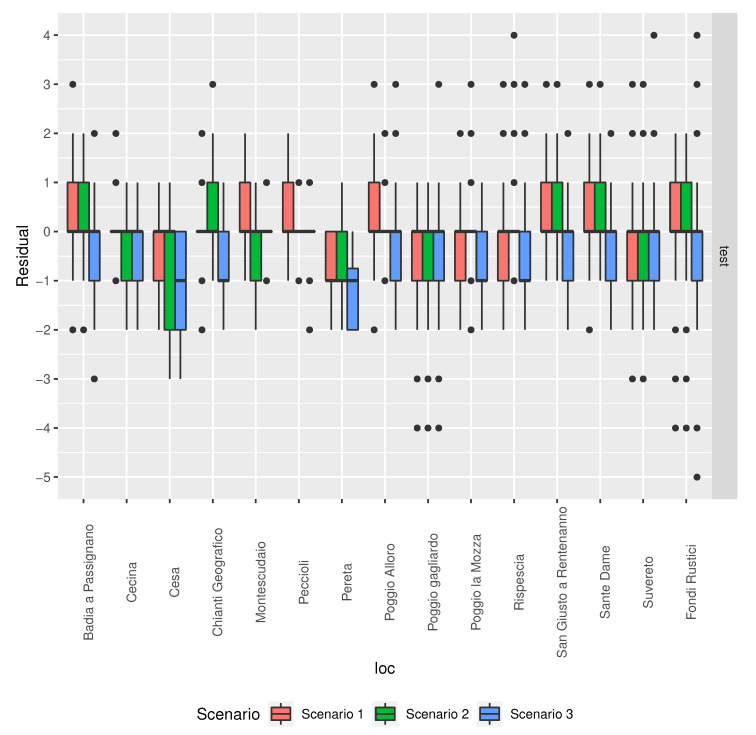
Comparison of the residuals by location for the different models’ in the scenarios described in Table 3.

Figure 13 shows that the distribution of the residuals differs by location. This would suggest that there is room for optimisation of the predictions by training local models. Now that satellite data are available for any location in the world, it would only be necessary to collect the BBCH observations for a specific location.

### 3.4. Optimisation of the Base Temperature

**Figure 14:**
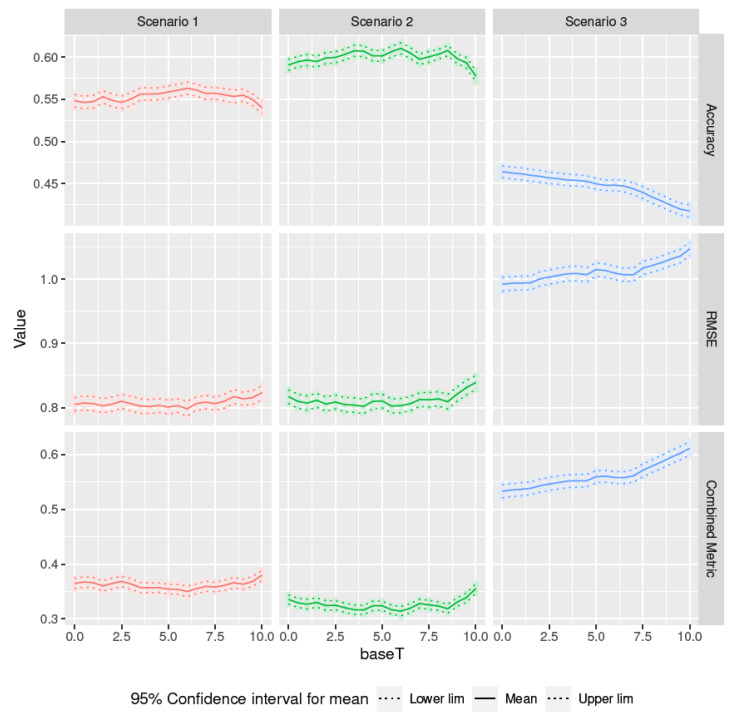
Base temperature optimisation for the scenarios described in Table 3: confidence intervals for Accuracy, RMSE, and combined metric.

The scenarios contemplated in this section are those described in Table 3. Figure 14 shows the confidence intervals obtained and Table 4 shows the optimal (minimum mean in the case of RMSE and combined metric, and maximum mean in the case of accuracy) base temperature values for each scenario and metric. Scenarios 1 and 3 obtain a single optimal value for all metrics, 6 °C for scenario 1 and 0 °C for scenario 3 (in this case, the same result as in [35]). In scenario 2, 6 °C maximizes the accuracy and minimizes the combined metric, but to minimize the RMSE the base temperature should be 4 °C.

## 4. Discussion

The results show that the temperatures derived from the ERA5 dataset and measured by weather stations provided similar GDD values (i.e., *GDD Tavg* and *GDD Allen*), as evidenced by the minor deviation from the 1:1 linear relationship. Small deviation was probably determined by local topography, such as elevation, which warrants further investigation. Oteros et al. [36] observed that altitude and percentage eastward slope were the most influential topographical factors affecting local olive tree phenology in southern Spain. Nevertheless, Copernicus’ ERA5 data were proved to be a valid alternative to calculate GDD, confirming that phenological modeling based on application of meteorological stations and reanalysis products may be extended to olive tree areas sharing the same plant phenological phases. This is an important finding, since weather station data are available only at a small number of locations and a globally available data source is necessary for regional predictions of phenological phases. Predicting the timing of developmental events is, indeed, critical for optimising crop productivity and production quality. Temperature is the main driver of tree phenology and, therefore, temperature data are essential for the development and application of phenophase model prediction.

The analysis of the performance of different feature subsets as predictors of plant developmental events and the predictive capability of machine learning models was carried out in three different scenarios, which correspond to the two different groups of predictors, ’Weather station data’ and ’ERA5’, and the final scenario, i.e., the baseline model. Both of the groups contain feature sets that include the DOY feature. DOY was not a good predictor, but the results show that, within the same scenario, the feature sets that contained DOY performed better than those without it. The best performing feature sets were (DOY, *GDD Allen*) in the ’Weather station data’ scenario and (DOY, *ERA5 GDD Tavg*) in the ’ERA5’ scenario, with the latter outperforming the former in terms of combined metric (in both mean and median), having clearly higher accuracy and only slightly higher RMSE. This is remarkable, because ERA5 data are more widely available than weather station data and *GDD Tavg* is quite faster and less resource intensive to compute than *GDD Allen* with data processing services, such as Google Earth Engine.

Regarding the selection of the best performing machine learning model for the best performing feature sets in the two scenarios, XGBoost (linear, with and without polynomial features) was the model with the highest accuracy and random forest (with and without polynomial features) that with the lowest RMSE, in both scenarios. When considering the combined metric, random forest was the best performing algorythm with the minimum mean, in both scenarios. Additionally, random forest had a lower mean combined metric in scenario 2 than in scenario 1. This opens new ground for developing phenology prediction models without the use of temperature data recorded at weather stationss. This has important practical consequences for implementations of machine learning approach to predict olive tree phenology. Therefore, we may infer that species-environment interactions can be projected in time and scaled in space at competitive costs and providing answers in a short time [37]. It must be pointed out that the training of machine learning algorithms requires rich datasets that are only rarely available.

In comparison with the baseline model, the selected machine learning models showed better accuracy. The baseline model was the only model with residuals of magnitude 5, and also the one with a significantly larger number of residuals of magnitude 4. All in all, the baseline model was the worst performing scenario, with more numerous and larger errors. Scenario 1, on the other hand, had lower accuracy than scenario 2, but most of its residuals were of magnitude 1 and had a lower number of residuals of magnitude greater than 1 than scenario 2. The baseline model had the largest errors for high DOYs (late timing), whereas for low DOYs (early timing) it underestimated the phenology phase. The baseline model underestimated the prediction for the first 16 phenological phases, while the models in ’Weather station data’ and ’ERA5’ made better predictions for these phases. Yet, the baseline model and models in scenarios 1 and 2 made errors of large magnitude for the last 4 phenological phases, often overestimating the prediction. Errors of the machine learning models and those of the baseline model were often in different directions. The results also showed that the distribution of residuals differed by location. Ensemble models combining the baseline model with models in the other scenarios trained with local data will probably optimize the predictions. Modeling that is based on simultaneous application of meteorological records and satellite images used as predictor variables to project phenological phases may allow for application of combined approaches [38] in all locations where BBCH observations are available.

Regarding the optimisation of the base temperature, scenario 1 and the baseline model resulted in a single optimal value for all metrics, 6 °C for scenario 1 and 0 °C for scenario 4. In the case of scenario 2, 6 °C maximized the accuracy and minimized the combined metric, though the base temperature should be 4 °C in order to minimize the RMSE. In the Mediterranean Basin, areas of olive tree cultivation appear to be particularly sensitive to climate change, and Global Circulation Models forecast further increases in temperature and reductions in rainfall with a significant impact on olive tree growth and shift of suitability zones [39]. The present approach for modeling phenological phases of the olive tree may provide reliable projections for future climatic conditions at the landscape level, but can also be potentially applicable to local circumstances for operational forecasting of crop productivity.

## 5. Conclusions

The differences in predicting the phenological phases of olive trees between ERA5-driven models and those based on weather station data were negligible. The accuracy obtained from data reanalysis using machine learning algorithms allowed us to improve the traditional approach for modelling olive tree phenology, generating reliable predictions. Because weather station data are not globally available, ERA5-derived data can be conveniently used as substitute for recorded data. This approach provides a valid alternative for modelling phenological events where weather station data are not available or where their network does not ensure a sufficient coverage of the interested area. However, the implementation of ERA5 reanalysis in decision support systems for real-time forecasting of olive tree phenological phases requires the integration with data acquisition from regional networks and meteorological stations, since there is a time lag between the release date of data and the current date (generally 2–3 months).

## Figures and Tables

**Table 1:** Description of the two different scenarios in which to select a ML model for phenology phase prediction.

Scenario	Data Group	Features
Scenario 1	Weather station data	DOY, GDD (ALLEN)
Scenario 2	ERA5	DOY, ERA5_GDD (Tavg)

**Table 2:** The selection of the best performing model for each selected feature combination following the minimum mean combined metric criterion.

Scenario	Data Group	Feature Set	Selected Model	Mean
Scenario 1	Weather station data	DOY, GDD (ALLEN)	Random forest	0.38
Scenario 2	ERA5	DOY, ERA5_GDD (Tavg)	Random forest	0.35

**Table 3:** Scenarios for comparing selected ML models to Agricolus’ baseline model.

Scenario	Data Group	Feature Set	Selected Model
Scenario 1	Weather station data	DOY, GDD (ALLEN)	Random forest
Scenario 2	ERA5	DOY, ERA5_GDD (Tavg)	Random forest
Scenario 3	Weather station data	GDD (Allen)	Agricolus baseline

**Table 4:** Optimal values for the base temperature by metric for the scenarios described in Table 3.

Scenario	Metric	Optimal Base Temperature
Scenario 1	Accuracy	6.00
Scenario 1	RMSE	6.00
Scenario 1	Combined Metric	6.00
Scenario 2	Accuracy	6.00
Scenario 2	RMSE	4.00
Scenario 2	Combined Metric	6.00
Scenario 3	Accuracy	0.00
Scenario 3	RMSE	0.00
Scenario 3	Combined Metric	0.00

## Abbreviations

The following abbreviations are used in this manuscript:

ECMWF	European Centre for Medium-Range Weather Forecasts
ERA5	ECMWF Reanalysis 5th Generation
BBCH	*Biologische Bundesanstalt, Bundessortenamt und Chemische Industrie*
GDD	Growing degree day
DOY	Day of year
DSS	Decision Support System
CART	Classification and regression trees
ANN	Artificial neural networks
GBM	Stochastic gradient boosting
XGBoost	Extreme gradient boosting
RF	Random forest
RMSE	Root mean square error
*GDD Tavg*	GDD calculated following the average temperature method using weather station data
*ERA5 GDD Tavg*	GDD calculated following the average temperature method using ERA5 data
*GDD Allen*	GDD calculated following the Allen method using weather station data
*ERA5 GDD Allen*	GDD calculated following the Allen method using ERA5 data
